# Propolis: A Smart Supplement for an Intracanal Medicament

**DOI:** 10.5005/jp-journals-10005-1459

**Published:** 2017-02-27

**Authors:** Rashmi Baranwal, Vijay Duggi, Alok Avinash, Alok Dubey, Sulabh Pagaria, Harsha Munot

**Affiliations:** 1 Student, Department of Pedodontics and Preventive Dentistry Rungta College of Dental Sciences & Research, Bhilai Chhattisgarh, India; 2 Head, Department of Pedodontics and Preventive Dentistry Rungta College of Dental Sciences & Research, Bhilai Chhattisgarh, India; 3 Reader, Department of Pedodontics and Preventive Dentistry Rungta College of Dental Sciences & Research, Bhilai Chhattisgarh, India; 4 Associate Professor, Department of Pedodontics and Preventive Dentistry, College of Dentistry, Jazan University, Jazan, Kingdom of Saudi Arabia; 5 Postdradugate Student, Department of Pedodontics and Preventive Dentistry Rungta College of Dental Sciences & Research, Bhilai Chhattisgarh, India; 6 Postdradugate Student, Department of Pedodontics and Preventive Dentistry Rungta College of Dental Sciences & Research, Bhilai Chhattisgarh, India

**Keywords:** Calcium hydroxide, Propolis, Propylene glycol.

## Abstract

**Introduction:**

One of the most important factors for successful endodontic therapy is root canal cleaning. The difficulty involved in eliminating microorganisms, as well as their residual presence, warrants the use of root canal dressings after bio-mechanical preparation.

**Aim:**

The aim of the study was to compare the diffusion ability between nonalcoholic calcium hydroxide-propolis paste, calcium hydroxide-saline paste, and calcium hydroxide-propylene glycol paste.

**Materials and methods:**

For this proposed study, single-rooted extracted permanent teeth were randomly divided into three groups to fill the canals: group I: Calcium hydroxide-propylene glycol paste, group II: Calcium hydroxide-saline paste, and group III: Calcium hydroxide-propolis paste. After complete filing of the root canal, the pH values of the solutions in the flasks are measured at an interval of 3, 24, 72, 168 hours.

**Results:**

After 168-hour interval, it was noticed that the mean pH obtained by calcium hydroxide-propolis paste was 10.54 (± 0.38), which was greater than calcium hydroxide-propylene glycol paste 9.70 (± 0.45) and calcium hydroxide-saline paste 9.16 (± 0.30) consecutively.

**Conclusion:**

The nonalcoholic calcium hydroxide-propolis paste used during the study was able to diffuse through the dentinal tubules. Thus, it can be used as a vehicle for calcium hydroxide.

**How to cite this article:** Baranwal R, Duggi V, Avinash A, Dubey A, Pagaria S, Munot H. Propolis: A Smart Supplement for an Intracanal Medicament. Int J Clin Pediatr Dent 2017; 10(4):324-329.

## INTRODUCTION

Bacterial assault has been the most common etiology of dental pulp injury. There are various routes of entrance of bacteria and bacterial products within the pulp.^1^

The principal aim in endodontics is elimination or reduction of microorganisms in the root canal system in order to achieve clinical success. Intracanal medicaments have been used as an adjunct in endodontics.^2^ Of all the medicaments, calcium hydroxide is the most preferred material for an intracanal dressing as it has got favorable antimicrobial action.^3^

Calcium hydroxide was introduced by Hermann in 1920. It is widely used in endodontics. It is a highly alkaline substance with a pH of 12.5. It has got wide range of properties, such as antimicrobial activity, inhibition of tooth resorption, and induction of repair by hard tissue formation.^4^

The success of calcium hydroxide paste as an intra-canal medicament depends upon its dissociation into calcium and hydroxyl ions. Due to the highly alkaline environment, most of the endodontic pathogens are unable to survive. To continue its efficacy, the hydroxyl ions should be able to diffuse in dentin and should remain in pulp so as to produce the pH level required to destroy the bacteria inside within the root canal and dentinal tubules.

Out of the various medications found, propolis has attracted attention as an antimicrobial agent. Global trends toward natural products have led to the stimulus for further research for the medical potential of propolis. Propolis has been used for thousands of years as a popular medicine. In dentistry, it has been used to control the oral microflora.

The action of hydroxyl ions on tissues and bacteria is responsible for the biological and antimicrobial properties of calcium hydroxide.^5^

The aim and objectives of this study are to evaluate the diffusion ability of ions from a nonalcoholic calcium hydroxide-propolis paste through dentinal tubules and to compare the diffusion ability between nonalcoholic calcium hydroxide-propolis paste, calcium hydroxide-saline paste, and calcium hydroxide-propylene glycol paste.

## MATERIALS AND METHODS

The present study was carried out in the Department of Pedodontics and Preventive Dentistry in association with Department of Microbiology, Rungta College of Dental Sciences & Research, Bhilai, Chhattisgarh, India. The research protocol was reviewed and approved by the Institutional Ethical Committee, Ayush Health Science University, Chhattisgarh, India. For this proposed study, a total of 120 single-rooted orthodontically extracted permanent teeth were selected, and the teeth with resorbed root were excluded for the study.

**Table 1:** **Table 1:** Analysis of variance for pH values for different groups

				*Sum of squares*		*Degrees of freedom*		*Mean square*		*F-value*		*Significance*	
3 hours		Between groups		2.226		2		1.113		6.607		0.002	
		Within groups		19.710		117		0.168					
		Total		21.936		119							
24 hours		Between groups		5.072		2		2.536		23.011		0	
		Within groups		12.894		117		0.110					
		Total		17.967		119							
72 hours		Between groups		8.612		2		4.306		37.354		0	
		Within groups		13.487		117		0.115					
		Total		22.100		119							
168 hours		Between groups		38.433		2		19.216		136.466		0	
		Within groups		16.475		117		0.141					
		Total		54.908		119							

The teeth were randomly selected and divided into three groups of 40 each as follows:

- *Group I (n = 40):* Calcium hydroxide-propylene glycol paste (prepared by mixing 1 gm of calcium hydroxide and 2 mL of propylene glycol).
- *Group II (n = 40):* Calcium hydroxide-saline paste (prepared by mixing 1 gm of calcium hydroxide and 1.5 mL of saline solution).
- *Group III (n = 40):* Calcium hydroxide-propolis paste (prepared by mixing 1 gm of calcium hydroxide and 2 mL of propolis without alcohol).

The prospective investigation was a randomized controlled trial with an experimental period of 7 days. Initially, 120 extracted teeth were kept in a 10% formaldehyde solution. Next, the soft tissues and dental calculus that remained adhered to the root were removed with dental scalers, after which the tooth is stored in saline solution. Then, the crowns were transversally sectioned with the carborundum disk at the cementoenamel junction level. Root canal length was measured with a rubber stop. When the file tip reached the apical foramen, the stop was leveled to the cervical edge of the root and the canal length was recorded. The working length was established by subtracting 1 mm from the total root canal length. Apical preparation is performed up to this limit, up to file #80, followed by a step back instrumentation up to file #120. The root canals were irrigated with distilled water throughout the instrumentation procedure. After instrumentation, a #30 file was inserted to the total working length for apical cleaning, and the root canal was filled with an ethylenediaminetetraacetic acid solution for 3 minutes. After this period, the root canals were rinsed with saline solution and dried with absorbent paper points. The teeth were randomly divided into three groups. After complete filling of the root canals, their openings were sealed with temporary cement. The apical foramen and root canal opening were sealed with epoxy cement. Next, the teeth were placed in containers with 50 mL of deionized water (pH = 6.17) and kept in an oven at 37°C, with 100% humidity. After 3, 24, 72, and 168 hours, the pH values of the solutions in the flasks were measured with a pH meter. Data obtained were entered into Microsoft Excel spreadsheet and then transferred to Statistical Package for the Social Sciences, version 16.0 software for the statistical analysis.

## RESULTS

The pH values were recorded and evaluated using one-way analysis of variance followed by *post hoc* Tukey’s highest significant difference test.

**Graph 1:**
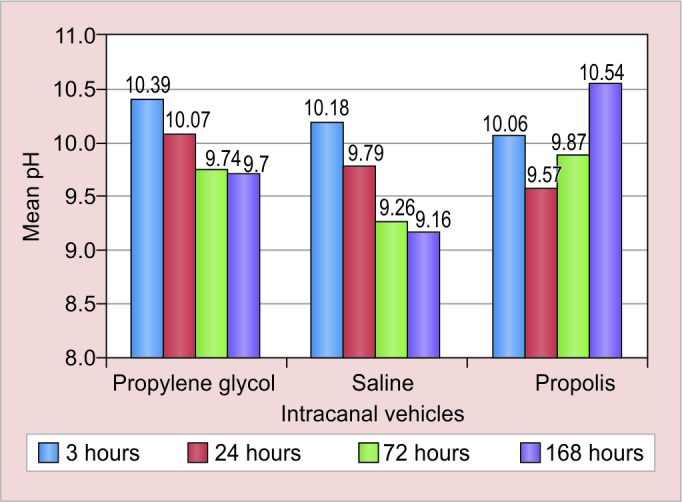
pH variation according to various study groups

**Graph 2:**
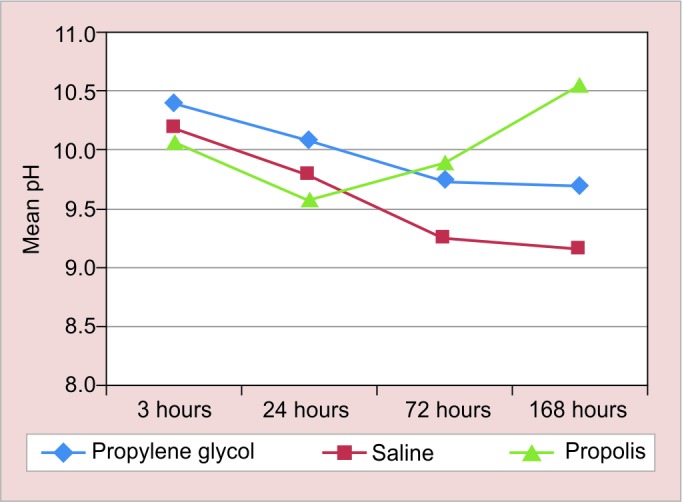
pH variation according to time interval

Postintervention comparison of the groups after 3, 24, 72 and 168 hours showed significant variation in all the three groups (p < 0.05) (Table 1, Graphs 1 and 2).

Hence, by conducting this study, it is proved that propolis can also be used as a vehicle for calcium hydroxide. On comparing the three vehicles, propolis may be a better indication as it possesses antimicrobial action.

## DISCUSSION

Endodontics has undergone a revolutionary change. The major goal of successful endodontic treatment is elimination of the bacteria from the root canal space. The outcome of endodontic therapy depends upon the reduction or elimination of bacteria. Chemomechanical preparation may be considered an essential step in the root canal disinfection. However, total elimination of bacteria is difficult to accomplish. Thus, by remaining within the root canal, intracanal medicaments help to eliminate surviving bacteria.^6^

The use of medicaments has been a routine practice for many years. In the past, numerous antimicrobial agents have been introduced as root canal medicaments. Since the introduction of calcium hydroxide for use in dentistry by Herman in 1920, this medicament has been reported to promote healing. It is a white odorless powder. It has got wide range of properties that include antibacterial activity, tissue dissolving ability, and induction of repair by hard tissue formation. The main benefit, i.e., antibacterial property is acquired by its high pH, which is 12.5.^7^

Among all the intracanal medicaments, calcium hydroxide was the most commonly used due to its alkaline nature. It was shown to be highly effective over the persistent root canal flora when canals were dressed for 7 days.^8^ Due to its superior activity and reduced cyto-toxicity, it has been considered as the material of choice. The hydroxyl ions produced due to its dissociation in aqueous fluids are believed to be responsible for alkaline nature, i.e., bactericidal. These hydroxyl ions induce lipid peroxidation which helps in destruction of phospholipids. The alkalinization causes destruction of the ionic bonds that form the tertiary structure of proteins. They also react with bacterial DNA and induce splitting of the strands.^9^

The delivery of calcium hydroxide powder alone is difficult or impossible, so it must be mixed with a liquid to facilitate its placement within the canal.^1^ Calcium hydroxide when mixed with different vehicles has shown the potential to release calcium and hydroxyl ions through cementum.^7^ The rate of dissociation into ions and diffusion through dentinal tubules is determined by the vehicles used. Thus, vehicles play an important role in the ionic dissociation.^5^

Lage Marques et al^10^ concluded that aqueous and viscous vehicles when mixed with calcium hydroxide seems to be more effective as compared with oily vehicles because aqueous and viscous vehicles reach higher pH at a faster rate and they remain stable for a longer period of time. Thus, calcium hydroxide must be associated to a vehicle for its use as an intracanal medicament.

Hence, the present study aimed at to evaluate the diffusion ability of ions from a nonalcoholic calcium hydroxide-propolis paste through dentinal tubules and to compare the diffusion ability between nonalcoholic calcium hydroxide-propolis paste, calcium hydroxide-saline paste, and calcium hydroxide-propylene glycol paste.

Propolis, renowned for its antimicrobial activity, was introduced into dentistry in 1996 by Krell.^11^ Propolis is a natural resinous mixture, which is produced by honeybees. It is also known as bee glue. It is used as a hive defense. Propolis is a complex mixture which are derived by plants and released by bees. Its composition varies according to geographical region.^12^ The raw propolis is composed of basically 50% resins, 30% waxes, 10% essential oils, 5% pollen, and 5% various organic compounds.^13-15^ It possesses a characteristic and pleasant aromatic smell. Depending upon its source and age, its color varies from yellow green to red and to dark brown.^16,17^ It consists of highly active bioflavonoids which have antimicrobial, antioxidant, and antiinflammatory properties. The antioxidant property of propolis is attributed to its radical scavenging ability, which was better than vitamin C and that the antiinflammatory property is due to the presence of caffeic acid phenethyl ester.^18^ It has been proved to be a less irritating solution^19^ and were effective in eliminating *Enterococcus faecalis.^20,21^* Propolis was also found to induce the production of high-quality tubular dentin.^22^ Propolis has been known to be a potent sensi-tizer, is allergenic, and may lead to eczematous contact dermatitis in some cases.^23^

De Rezende et al^24^ evaluated two propolis paste associated with calcium hydroxide with and without alcohol. They concluded that both the pastes displayed antimicrobial action, but the nonalcoholic paste produced greater inhibition halos as compared with the paste containing alcohol. That is why in the present study, nonalcoholic calcium hydroxide-propolis paste is used.

Another vehicle which is used is propylene glycol (1,2-propanediol), a dihydric alcohol. It was suggested by Laws in 1962 for its possible use as a vehicle in endodontics.^25,26^ Propylene glycol is a colorless liquid with mild acrid smell and sweet taste. Olitzky^27^ stated that concentrated solution of propylene glycol showed marked germicidal efficiency. So, it can be used as a vehicle which has the potential for preventing and treating microbial infections. Bhat and Walwekar,^25^ Thomas et al^28^ stated that as compared with other commonly used vehicles for intracanal medicaments, propylene glycol has been found to be less cytotoxic and also possesses antibacterial properties that are highly beneficial in the endodontic treatment. Fava and Saunders^29^ stated that propylene glycol possesses hygroscopic properties that allow absorption of water. This resulted in a sustained release of the intracanal medicament for prolonged period of time.

In the present study, single-rooted orthodontically extracted permanent teeth were selected and the experiment was carried out. Based on the experiment, it was concluded that propolis can also be used as a vehicle for calcium hydroxide as it possesses the ability to diffuse through dentinal tubules. It may be a better indication as compared with other intracanal vehicle as it adds to the antimicrobial action of calcium hydroxide.

Montero and Mori^5^ carried out a study on 36 extracted single-rooted bovine teeth and concluded that calcium hydroxide-propolis paste also diffused through the dentinal tubules and reach the external root surface. They also stated that the viscous consistency of propolis also favored the level of diffusion.

Ahangari et al^30^ investigated the antibacterial efficacy of propolis against *E. faecalis* compared with calcium hydroxide and concluded that antimicrobial activity of propolis against *E. faecalis* species was comparable with that of calcium hydroxide at different time intervals. Therefore, it can be used as an alternative natural material for disinfection of canals during endodontic treatment.

Similarly, a study was conducted by Jahromi et al.^2^ They evaluated and compared colony-forming units (CFUs) and minimum inhibitory concentrations (MICs) of calcium hydroxide and propolis as intracanal medicaments. They observed that MICs and CFUs of propolis were dramatically less than calcium hydroxide. They selected *E. faecalis* for their study, since it was a challenge to overcome this organism in case of periapical infections. They stated that calcium hydroxide showed a moderate antimicrobial efficacy against *E. faecalis,* while propolis showed significant efficacy in killing *E. faecalis.*

The use of propolis was recommended in the root canal fillings by the studies conducted by Russians (1990) because it possesses anesthetic and bone-regenerating properties. In the same year, studies were conducted by the Romanians. They stated that propolis seems to be effective as a pulp capping agent, thereby increasing the production of dentin and remineralization.^31^

Another vehicle which is used in this study is pro-pylene glycol which also showed the ability to diffuse through dentinal tubules. When the pH variation was compared with the other two vehicles, i.e., saline and propolis, then it was seen that calcium hydroxide-propylene glycol paste provided higher amount of hydroxyl ion release as compared with saline, while is lower as compared with propolis.

Cruz et al^32^ evaluated and compared penetration of propylene glycol and distilled water into root dentin and concluded that propylene glycol allowed dye to exit faster through the apical foramen. The area and depth of dye penetration with propylene glycol was significantly greater than with distilled water. They stated that the high surface tension of distilled water may have delayed the penetration through dentinal tubules; while in case of propylene glycol, already it is viscous as compared with distilled water but has got low surface tension, so this gives the advantage of being penetrable through dentinal tubules.

Chua et al^33^ investigated the antifungal activity of propolis, triple antibiotic paste (TAP), 2% chlorhexidine gel, and calcium hydroxide with propylene glycol on *Candida albicans*-infected root canal dentinal tubules at two different depths (200 and 400 μm) and two time intervals (days 1 and 7). They concluded that propolis was less effective than TAP, 2% chlorhexidine gel, and calcium hydroxide with propylene glycol against *C. albicans* on day 1 at 400 μm deep inside the dentinal tubules, but equally effective after 7 days at both depths. While in the present study, propolis showed the best results followed by propylene glycol and then by saline at the end of 7 days. In their study they stated that the reason behind the slower penetration of propolis at day 1 may be due to the slower rate of penetration in the dentinal tubules. The same reason could also stand true for the present study. They also stated that the antifungal action of propolis is due to the presence of flavonoids and phenolic acids which interact with the cellular sulfhydryl compounds on the cell wall, damage the integrity of the yeast cell wall, and result in detachment of fungal cell wall and reduction of germ tube formation and hyphal length. In addition, the antiinflammatory and antioxidant properties of propolis further enhances the healing of the periapical tissue.^34,35^

In the present study, at 168-hour interval, the pH level was highest in case of propolis, which may be due to the viscous consistency of propolis. Apart from this, the components present in this solution do not impair or prevent the dissociation of calcium hydroxide.^5^

Shruthi and Suma^36^ stated that caution should be taken for the people who are allergic to pollen, including asthmatic patients, pregnant women, and patients allergic to bee stings.

This is one of the major drawbacks associated with the use of propolis, which cannot be overlooked in any circumstances and there is need for further studies to be carried out in regard to application of propolis.

In the present study at an interval of 3 hours, the mean pH obtained by calcium hydroxide-propylene glycol paste was highest, followed by saline group and then by propolis group. The reason behind this may be due to the low surface tension of propylene glycol, which gives an advantage to be highly penetrable through the dentinal tubules.^32^

At 24-hour interval, the mean pH obtained by calcium hydroxide-propylene glycol and calcium hydroxide-propolis was found to be almost same, while the pH value of saline group was found to be lowered. The reason behind the drop in pH in saline group may be due to the formation of insoluble calcium carbonate crust, which blocks the dentinal tubules, resulting in decrease in the pH and stabilization of the ionic release.^37^

At 72- and 168-hours intervals, it was noticed that the mean pH obtained by calcium hydroxide-propolis paste was greater than calcium hydroxide-propylene glycol paste and calcium hydroxide-saline paste consecutively. Hence, it was proven that calcium hydroxide-propolis paste has better diffusion ability as compared with the other two experimental pastes. Therefore, propolis can also be used as a vehicle for calcium hydroxide.

After 168-hour interval, it was noticed that the mean pH obtained by calcium hydroxide-propolis paste was 10.54 (± 0.38), which was greater than calcium hydroxide-propylene glycol paste 9.70 (± 0.45) and calcium hydroxide-saline paste, 9.16 (± 0.30) consecutively. Hence, it was proven that calcium hydroxide propolis paste has better diffusion ability as compared with the other two experimental pastes. Therefore, propolis can also be used as a vehicle for calcium hydroxide.

However, further *in vivo* and *in vitro* studies are required to investigate the biocompatibility of this paste and thus can confirm the further use of propolis without alcohol as a vehicle for calcium hydroxide.

## CONCLUSION

The nonalcoholic calcium hydroxide-propolis paste used during the study was able to diffuse through the dentinal tubules. Thus, it can be used as a vehicle for calcium hydroxide. After 168 hours, all the experimental group showed the ability of diffusion in the dentin and also showed the potential to alkalize the external root surface.
